# Identification of a novel biomarker candidate, a 4.8-kDa peptide fragment from a neurosecretory protein VGF precursor, by proteomic analysis of cerebrospinal fluid from children with acute encephalopathy using SELDI-TOF-MS

**DOI:** 10.1186/1471-2377-11-101

**Published:** 2011-08-12

**Authors:** Takeshi Asano, Shinya Koizumi, Atsushi Takagi, Takayuki Hatori, Kentaroh Kuwabara, Osamu Fujino, Yoshitaka Fukunaga

**Affiliations:** 1 Department of Pediatrics, Nippon Medical School, Chiba Hokusoh Hospital, 1715 Kamagari, Inzai City, Chiba 270-1694, Japan

## Abstract

**Background:**

Acute encephalopathy includes rapid deterioration and has a poor prognosis. Early intervention is essential to prevent progression of the disease and subsequent neurologic complications. However, in the acute period, true encephalopathy cannot easily be differentiated from febrile seizures, especially febrile seizures of the complex type. Thus, an early diagnostic marker has been sought in order to enable early intervention. The purpose of this study was to identify a novel marker candidate protein differentially expressed in the cerebrospinal fluid (CSF) of children with encephalopathy using proteomic analysis.

**Methods:**

For detection of biomarkers, CSF samples were obtained from 13 children with acute encephalopathy and 42 children with febrile seizure. Mass spectral data were generated by surface-enhanced laser desorption/ionization time-of-flight mass spectrometry (SELDI-TOF MS) technology, which is currently applied in many fields of biological and medical sciences. Diagnosis was made by at least two pediatric neurologists based on the clinical findings and routine examinations. All specimens were collected for diagnostic tests and the remaining portion of the specimens were used for the SELDI-TOF MS investigations.

**Results:**

In experiment 1, CSF from patients with febrile seizures (n = 28), patients with encephalopathy (n = 8) (including influenza encephalopathy (n = 3), encephalopathy due to rotavirus (n = 1), human herpes virus 6 (n = 1)) were used for the SELDI analysis. In experiment 2, SELDI analysis was performed on CSF from a second set of febrile seizure patients (n = 14) and encephalopathy patients (n = 5). We found that the peak with an m/z of 4810 contributed the most to the separation of the two groups. After purification and identification of the 4.8-kDa protein, a 4.8-kDa proteolytic peptide fragment from the neurosecretory protein VGF precursor (VGF4.8) was identified as a novel biomarker for encephalopathy.

**Conclusions:**

Expression of VGF4.8 has been reported to be decreased in pathologically degenerative changes such as Alzheimer's disease, amyotrophic lateral sclerosis (ALS), frontotemporal dementia, and encephalopathy. Thus, the VGF4.8 peptide might be a novel marker for degenerative brain conditions.

## Background

Acute encephalopathy is characterized by sudden onset of high fever, lethargy, convulsions, and loss of consciousness, with poor prognoses associated with virus infections including causative viruses such as the influenza virus and other etiologies [1-3]. Given that encephalopathy leads to rapid deterioration and a poor prognosis, early intervention is essential in order to prevent progression of the disease and neurologic complications. However, in the acute period, true encephalopathy cannot easily be differentiated from febrile seizures, especially febrile seizures of the complex type [4-7]. Thus, an early diagnostic marker has been sought to enable early intervention in the disease.

Surface-enhanced laser desorption/ionization time-of-light mass spectrometry (SELDI-TOF MS) technology is currently applied in many fields of the biological and medical sciences, and has been extensively reviewed [8]. SELDI is a chromatography-based mass spectrometry (MS) platform in which proteins are selectively absorbed onto a chemically modified ProteinChip (Bio-Rad Laboratories, Inc., Hercules, CA, USA) array surface prior to the addition of an energy-absorbing matrix solution. The absorbed proteins are subjected to a pulsed laser beam to produce gas-phase ions that are accelerated into a field-free flight tube and separated according to their mass-dependent velocities (mass/charge: m/z). The ability of the selective array surfaces to retain subsets of the proteome allows the analysis of complex biological specimens such as serum, cell lysates, and cerebrospinal fluid. In the present study, SELDI-TOF MS was employed to identify a possible biomarker in cerebrospinal fluid for acute encephalopathy and a proteolytic peptide fragment from the neurosecretory protein VGF precursor was identified as a novel biomarker for encephalopathy.

## Methods

### Definitions of acute encephalopathy and febrile seizures

Because acute encephalopathy is a generic term for acute brain dysfunction usually preceded by infection, encephalopathy related to infection as acute encephalopathy was not excluded [1,2].

Acute encephalopathy: The criteria for diagnosis of encephalopathy were 1) impaired consciousness, 2) signs of increased intracranial pressure due to brain edema, 3) convulsions and seizures, 4) slow activity on electroencephalography lasting more than 24 h after acute onset, and 5) no bacteria or fungi on CSF culture [1,2,9].

Febrile seizures: Febrile seizures were defined as 1) seizures with fever and impaired consciousness lasting less than 24 h without neurological sequelae, 2) usually occurring between 3 months and 5 years of age, and 3) without evidence of intracranial infection or defined cause [4-7,9].

### Subjects

Two studies were performed on cerebrospinal fluid (CSF) from patients at the Department of Pediatrics, Nippon Medical School Chiba Hokusoh Hospital. In an initial study (experiment 1) for discovering the new, novel biomarkers, CSF from febrile seizure subjects (n = 28) and patients with encephalopathy (n = 8), including influenza encephalopathy (n = 3), encephalopathy due to rotavirus (n = 1), human herpes virus 6 (n = 1) were used for the SELDI analysis. In a second study (experiment 2) for validating the markers, SELDI analysis was performed on CSF from a second set of febrile seizure subjects (n = 14) and encephalopathy patients (n = 5). Diagnosis was made by at least two pediatric neurologists based on clinical findings, routine examinations described above, and lumber puncture performed at the onset of illness.

All CSF specimens were collected for diagnostic tests and the remaining portions of the specimens were used for SELDI-TOF MS investigation. Following collection, samples were centrifuged at 1500 rpm for 5 min to remove cells, divided into aliquots and immediately frozen on dry ice and stored at -80°C. The Institutional Review Board at Nippon Medical School, Chiba Hokusoh Hospital approved the collection and investigation of samples and written informed consent was obtained from all subjects.

### Proteomic technology

SELDI-TOF MS is a system that enables rapid protein profiling, identification, and characterization from crude biological samples by selective capture of subclasses of proteins with specific physical or biochemical characteristics. The molecular weight as well as the quantity of individual proteins absorbed on each ProteinChip array is then directly assessed by a time-of-flight mass spectrometer, generating quantitative protein mass profiles for individual CSF specimens. Comparative protein expression profile analysis highlights any CSF protein species which are aberrantly expressed in the CSF of encephalopathy patients compared to febrile seizure subjects.

Before analysis of the CSF, experiments were designed to optimize the ProteinChip array conditions for the SELDI-TOF MS proteomics tests. Three chip types, Q10 (strong anion exchange surface), CM10 (weak cation exchange surface), and IMAC30 (metal binding surface) (Bio-Rad), were tested along with a variety of binding/washing conditions, including pH 4.5, 6.5, and 8.0 binding/washing buffers. The ProteinChip array condition that produced optimal results was the Q10 ProteinChip array with 50 mM Tris-HCl, pH 8.0 binding and wash steps, and was therefore used in this study.

For all experiments, a 96-well ProteinChip cassette-compatible bioprocessor was used. The ProteinChip array spot surface was equilibrated with wash buffer (50 mM Tris-HCl, pH 8.0) for 10 min prior to use. CSF specimens were centrifuged and fresh aliquots of the CSF specimens were stored at -80°C. For analysis, these samples were slowly thawed on ice, centrifuged (10000 × g, 4°C, 15 min). Equal amounts of CSF protein (1.5 μg) diluted with 9 volumes of washing buffer were applied to each ProteinChip array spot. ProteinChip arrays were then incubated in a humidity chamber at room temperature for 60 min with shaking at 200 rpm. Unbound proteins were removed by washing three times for 5 min with binding buffer and then briefly washed with HPLC grade water. An energy absorbing molecule (EAM), saturated sinapinic acid (SPA), in a solution containing 50% acetonitrile/5% trifluoroacetic acid, was applied to the ProteinChip array spot surface. The arrays were air dried and proteins captured on individual spots were evaluated using the PBSIIc ProteinChip reader (Bio-Rad). The ProteinChip reader was externally calibrated with protein and peptide standards (Bio-Rad) containing human angiotensin I (1296.5 Da), Fibrinopeptide B (1570.6 Da), porcine Dynorphin A [209-225] (2147.5 Da), human ACTH [1-24] (2933.5 Da), human β-endorphin [61-91] (3465.0 Da), bovine insulin (5733.6 Da), bovine Ubiquitin (8564.8 Da), bovine cytochrome-C (12230.9 Da), bovine superoxide dismutase (15591.4 Da), equine myoglobin (16951.5 Da), bovine β-lactogloburin A (18363.3 Da), horseradish peroxidase (43240.0 Da), bovine albumin (66410 Da), and chicken conalbumin (77490.0 Da) bovine IgG (147300.0 Da). External calibration provided a 0.1% mass accuracy. The ionized proteins were detected and their molecular mass/charge (m/z) ratios determined using time-of-flight mass spectrometry (TOF-MS) analysis with a detection range m/z of 1,570-12,230 for low molecular range, 5,733 to 43,240 for mid range and 16,951-147,300 for high molecular range. The setting for cluster formation at the first pass was set as S/N of 20% in all spectra, and for the second pass S/N was set to 2%. The cluster mass window was set to 0.3%. In one experiment, 713 peaks were identified. The protein concentration in CSF was determined using Bradford methods (Bio-Rad) in a procedure consistent with the manufacturer's instructions.

Protein peaks were analyzed with the ProteinChip Data Manager software version 3.0 (Bio-Rad). Each study was repeated at least twice, with baseline subtraction, spectrum normalization, and peak detection performed using the ProteinChip software.

### Data collection and statistical methods

All spectra were externally mass calibrated and peak intensities were normalized using total ion current. The mean peak intensity of the duplicates was used for statistical analysis. Statistical analysis was also performed using the Kruskal-Wallis H test. When differences were significant, the Mann-Whitney *U *test was used to determine the significance of differences between each group. Uncorrected *p *values were corrected by multiplying them by the number of comparisons (Bonferroni-Dunn correction) to calculate corrected *p *values.

#### Purification of the 4.8-kDa protein

Methodology for purifying the 4.8-kDa protein was developed for on-chip analysis and also scaled up to column chromatography. First, conditions of capture were defined using a combination of various binding/washing buffers (100 mM sodium acetate, pH 4.0 and 5.0; 50 mM sodium phosphate, pH 6.0 and 7.0; and 50 mM Tris-HCl, pH 8.0 and 9.0) and anion exchange (Q10) and cation exchange (CM10) ProteinChip arrays. The 4.8-kDa protein was captured by Q10 ProteinChip arrays with the pH 4.0-8.0 conditions. Then, 500 μl of a CSF sample was mixed with an equal volume of 50 mM sodium acetate buffer (pH 4.0) containing 0.1% octyl glucoside (Wako Chemicals, Osaka, Japan). A 100 μl aliquot of the mixture was passed through an anion exchange column (Q Sepharose Fast Flow, GE Healthcare, Tokyo, Japan) and eluted with 50, 100, 200, 300, 500, and 1000 mM NaCl in 50 mM sodium acetate buffer, pH 4.0 containing 0.1% octyl glucoside. Each reverse phase fraction was analyzed for the presence of the 4.8-kDa peak using NP20 ProteinChip arrays (Bio-Rad). Protein species in reverse phase fractions were concentrated using micro-C18 ZipTip (Millipore, Billerica, MA, USA) following manufacturer's instructions, and reduced and alkylated using iodoacetamide.

##### Peptide Mass Fingerprint (PMF) analysis of the 4.8-kDa peak

The 4.8-kDa peptide was subjected to peptide mass fingerprinting (PMF) using ProteinChip arrays. Briefly, purified species were applied onto a normal phase ProteinChip array (NP20) and reduced with 5 mM DTT/10 mM NH_4_HCO_3_, (pH 8.0) at 70°C. Then, 2 μg/ml of trypsin (Promega, Tokyo, Japan) in 10 mM NH_4_HCO_3 _(pH 8.0) was applied and incubated for 2 h at 37°C. Digested peptides were detected using the ProteinChip reader and the data were analyzed using the Mascot search engine.

##### Identification of the 4.8-kDa peptide by MS/MS analysis

For MS/MS analysis, the purified protein was digested overnight with trypsin at 37°C, desalted with a ZipTip (Millipore) eluted with 2 μl of an aqueous acetonitrile solution (containing 50% acetonitrile (v/v) and 0.1% formic acid), and loaded onto a GlassTip in preparation for quadrupole time-of-flight (Q-TOF) MS analysis. Q-TOF analysis was performed using a MALDI-QSTAR mass spectrometer (Applied Biosystems, Tokyo, Japan) operated in positive mode. The ions corresponding to the tryptic digest peptides from the 4810 m/z species were selected for subsequent collision-induced dissociation, and the resultant fragment ions were analyzed. The resulting MS/MS spectral information was submitted to the Mascot search engine for identification. Among the analyzed peaks, the m/z 1907 peak generated a good fragmentation pattern for the Mascot search. Search parameters and settings for the Mascot identification were as follows: database (NCBInr), taxonomy (Homo sapiens), enzyme (SemiTrypsin), fixed modification (none selected), variable modification (Carbamidomethyl (C)), peptide mass tolerance (± 0.3 Da), peptide tolerance (± 0.3 Da), MS/MS tolerance (± 0.15 Da), peptide charge (+1), monoisotopic and max one missed cleavages. Sixty one matching the ion was identified as gi|17136078, a neurosecretory protein VGF precursor [*Homo sapiens*] with score of 61 (score > 49 indicates identity) and gi|2244659, a neuro-endocrine specific protein VGF [*Homo sapiens*] with score of 61 (score > 49 indicates identity).

## Results

### Patient demographic analysis

For experiment 1, CSF samples were analyzed both from patients with febrile seizure (n = 28) and with encephalopathy (n = 8). There were no statistical differences between the children with acute encephalopathy and with febrile seizure in mean patient age, CSF cell number, total protein, or total sugar content (Table 1). For experiment 2, there were also no statistical differences in mean patient age, CSF cell number, total protein, or sugar content between the patient groups (Table 1).

**Table 1:** Demographics of subjects in CSF Experiment 1 and Experiment 2 sample sets

	Age (months)	Gender (M/F)	Number of cells in CSF (/ml)	Total protein in CSF (mg/dl)	Total sugar in CSF (mg/dl)
**Experiment 1**					
**Febrile seizure** **(n = 28)**	29.8 ± 24.9	20/8	1.2 ± 1.1	18.7 ± 8.0	84.5 ± 16.5
**Encephalopathy** **(n = 8)**	46.4 ± 33.4	4/4	1.2 ± 1.0	22.9 ± 11.5	95.0 ± 43.4
**Experiment 2** **Febrile seizure** **(n = 14)**	44.1 ± 40.6	10/4	1.5 ± 1.5	18.8 ± 4.1	84.1 ± 14.3
**Encephalopathy** **(n = 5)**	48.4 ± 38.5	3/2	3.0 ± 2.3	16.7 ± 6.0	76.3 ± 19.4

M/F: Male/Female.

### Experiment 1: Changes of CSF protein/peptide profiles in febrile seizure patients compared to encephalopathy patients

**Figure 1:**
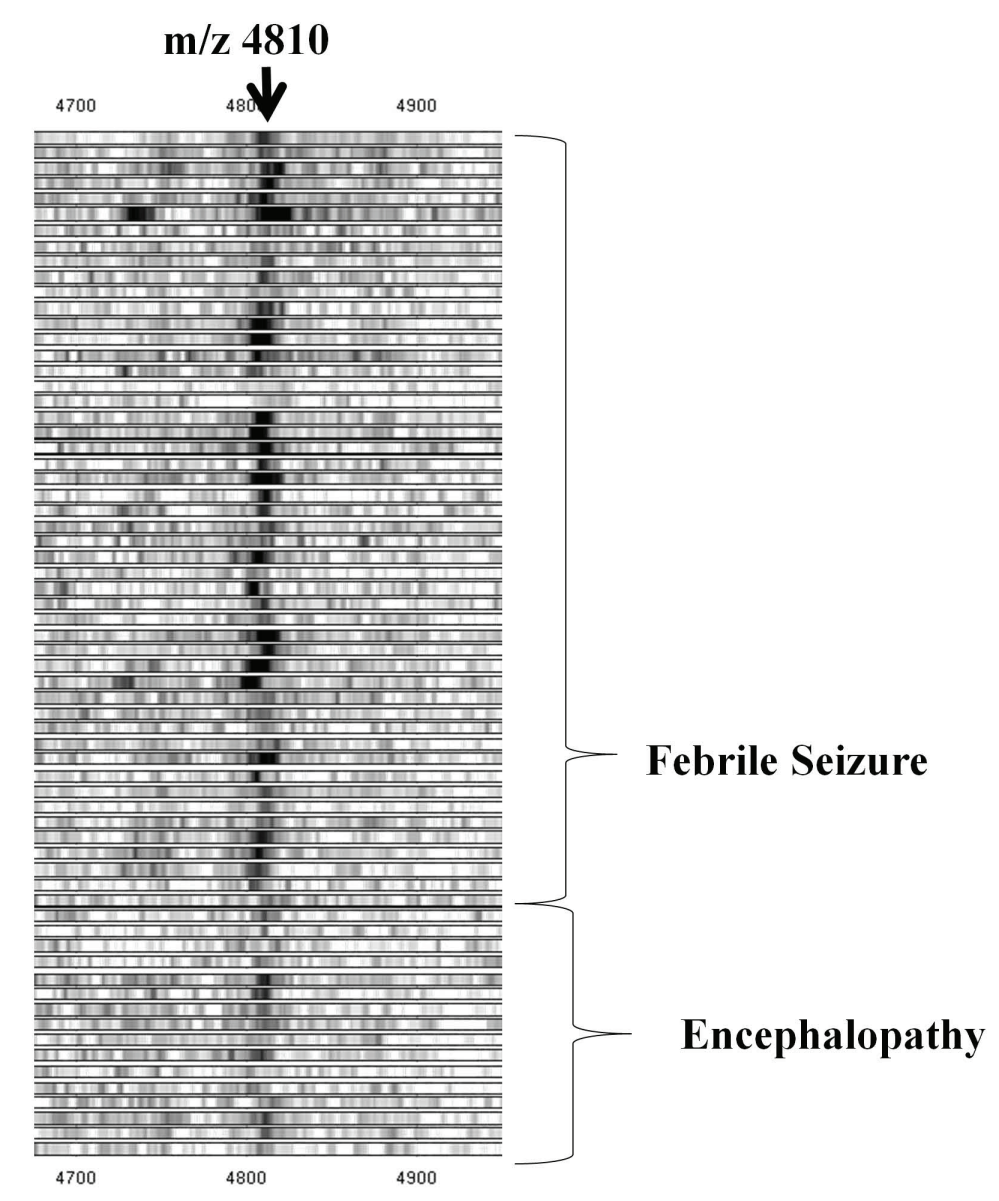
**Gel view of SELDI-TOF MS proteomic spectra of CSF from patients with febrile seizure and patients with encephalopathy**. Protein/peptide profiles of CSF samples were examined from 28 febrile seizure patients and eight encephalopathy patients using SELDI-TOF mass spectrometry. Because samples were measured in duplicate, a set of two gel views from one patient were provided.

**Figure 2:**
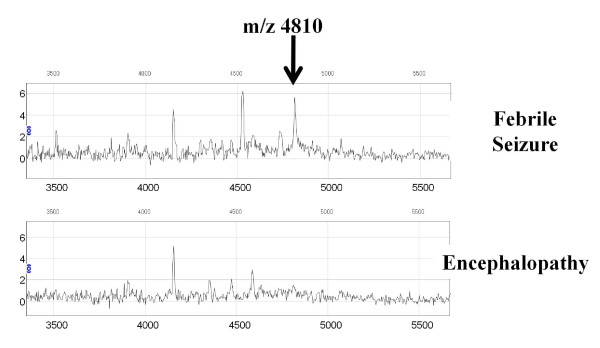
**Representative data of SELDI-TOF MS proteomic spectra of CSF from patients with febrile seizure and patients with encephalopathy**.

**Figure 3:**
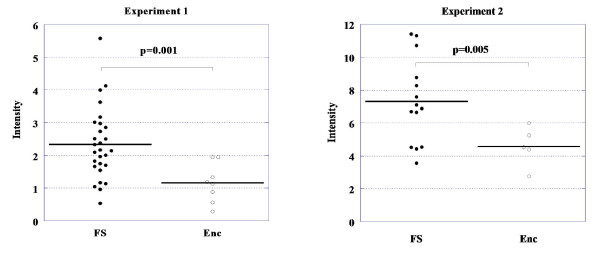
**SELDI-TOF MS intensities of the m/z 4810 peak from experiments 1 and 2**. **Experiment 1: **The mean intensity of the m/z 4810 peak was reduced by 39% (p = 0.001) in encephalopathy patients compared to patients with febrile seizures. **Experiment 2: **The mean intensity of the m/z 4810 peak was reduced by 41% (p = 0.005) in encephalopathy patients compared to patients with febrile seizures. FS: febrile seizure, Enc: encephalopathy.

In the first set of experiments (experiment 1), protein/peptide profiles of CSF samples were examined from 28 febrile seizure patients and eight encephalopathy patients (Table 1) using SELDI-TOF mass spectrometry. Fifteen peaks showed clear differences between febrile seizure patients and encephalopathy patients (p < 0.05, data not shown). Among these, six peaks had higher intensity in patients with febrile seizures compared to encephalopathy patients, and nine peaks were higher in patients with encephalopathy compared to febrile seizure patients (data not shown). Among these peaks, the peak at m/z 4810 contributed the most to the separation of the two groups (Figures 1, 2 and 3). Using the Kruskal-Wallis H test and the Mann-Whitney *U *test to compare the mean signals from both groups, the mean intensity of the m/z 4810 peak was found to be reduced by 39% (p = 0.001).

### Experiment 2

**Figure 4:**
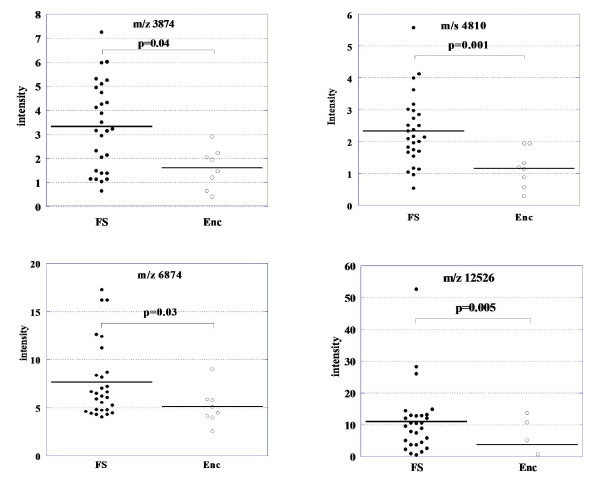
**Scatter plots for differentially expressed peaks in experiments 1 and 2**. Four peaks (m/z 3874, 4810, 6874, and 12526) showed significant difference in the mean intensity between encephalopathy patients and febrile seizure patients.

In experiment 2, the 15 candidate biomarkers from the experiment 1 were analyzed using an independent, albeit smaller, set of CSF samples from 14 febrile seizure patients and five encephalopathy patients (Table 1). Among the 15 peaks which were identified in experiment 1, 14 peaks were identified in experiment 2, but only four peaks showed statistically significant differences (Figure 4 Table 2). Again, as in experiment 1, the peak with an m/z of 4810 contributed the most to the separation of the two groups (Figure 1, 2, 3 and 4). Using the Kruskal-Wallis H test and the Mann-Whitney *U *test to compare the mean peak intensity from both groups, the mean intensity of the m/z 4810 peak was reduced by 41% (p = 0.005).

**Table 2:** Differentially expressed peaks

Peak (m/z)	Protein Chip Array Type	Level in encephalopathy	p-value of encephalopathy vs. febrile seizure (Experiment 1)	p-value of encephalopathy vs. febrile seizure (Experiment 2)
**3874**	Q10	Low	0.04	0.05
**4810**	Q10	Low	0.001	0.005
**6874**	CM10	High	0.03	0.01
**12526**	CM10	Low	0.005	0.001

### Identification of the 4.8-kDa CSF peptide distinguishing patients with febrile seizure from patients with encephalopathy

**Figure 5:**
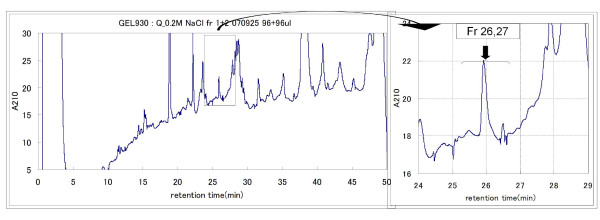
**Purification of the 4.8-kDa biomarker candidate using HPLC**. The 4.8-kDa protein eluted from the Q Sepharose Fast Flow anion exchange column in the 100 and 200 mM NaCl fractions. The 200 mM NaCl fraction eluted from the Q Sepharose Fast Flow anion exchange column was further purified by reverse phase HPLC.

**Figure 6:**
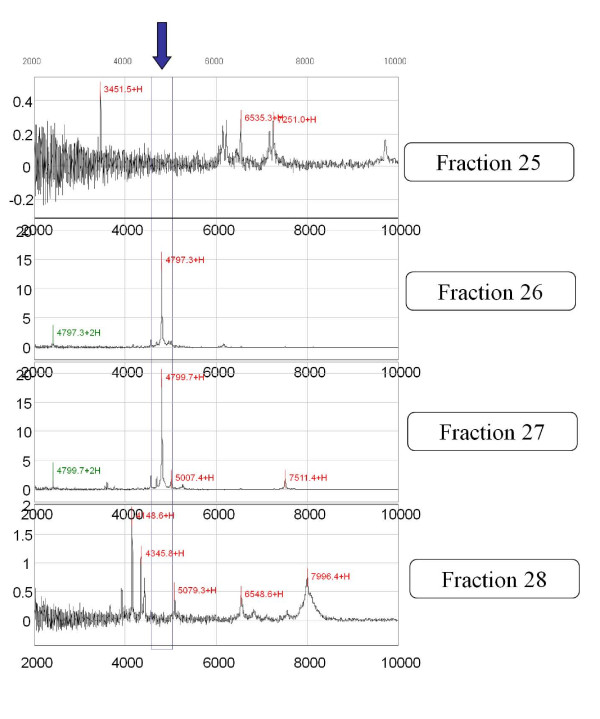
**Confirmation of the single peak in the 2000 to 10,000 Da range**. Each reverse phase fraction was analyzed by SELDI using NP20 ProteinChip arrays to detect the 4.8-kDa peak. It was confirmed that fractions 26 and 27 contained a predominant peak at 4.8 kDa in the 2000 to 10,000 Da range using NP20 ProteinChip arrays and SELDI analysis.

**Figure 7:**
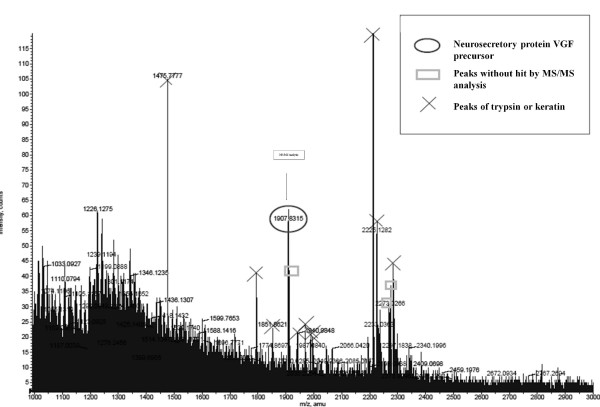
**MS analysis of the trypsin digest of the purified protein**. The purified protein was digested overnight with trypsin at 37°C and desalted in preparation for Q-TOF MS analysis. Q-TOF analysis was performed using a MALDI-QSTAR. The ions corresponding to the tryptic digest peptides from the 4810 m/z species were selected for subsequent collision-induced dissociation and the resultant fragment ions were analyzed. The resulting MS/MS spectral information was submitted to the Mascot search engine for identification. Among the analyzed peaks, the m/z 1907 peak generated a good fragmentation pattern for the Mascot search.

**Figure 8:**
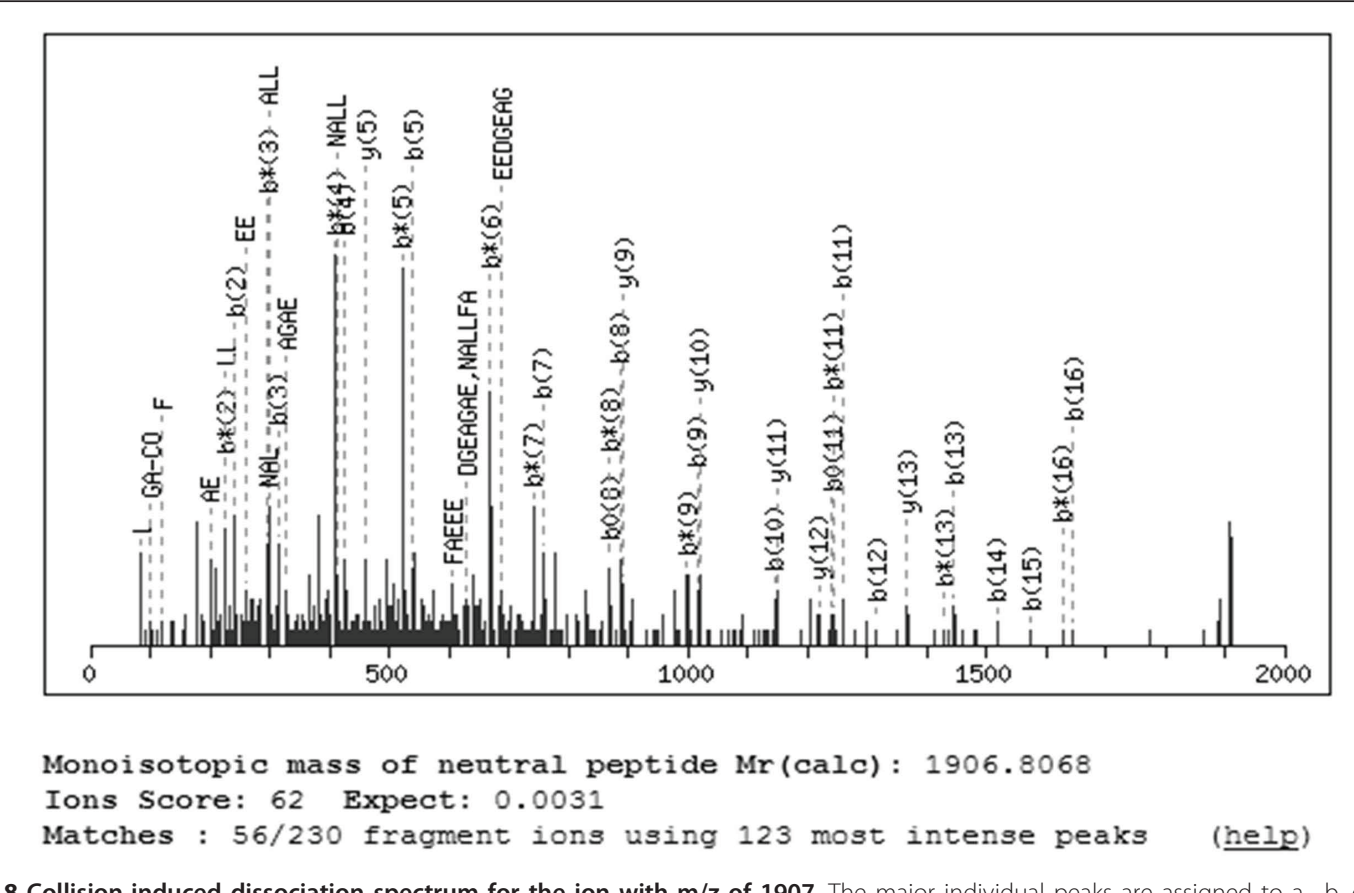
**Collision-induced dissociation spectrum for the ion with m/z of 1907**. The major individual peaks are assigned to a-, b, or y-type ions of di- or tri-peptide fragments.

**Figure 9:**
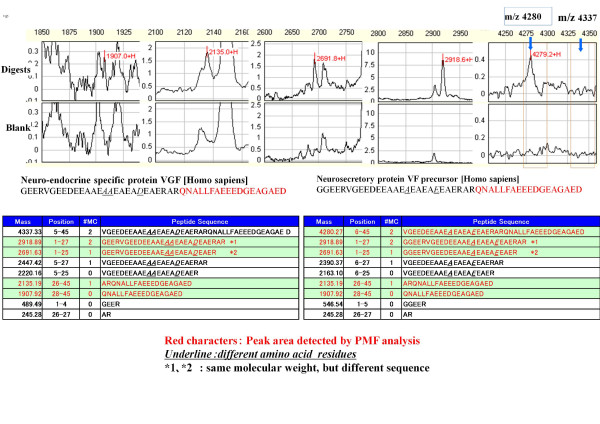
**Peptide Mass Fingerprint (PMF) analysis of the purified 4.8-kDa protein**. PMF analysis was used to distinguish between the two protein candidates identified by MS/MS. Spectrum data and sequences were obtained from the peptides digested by trypsin. The MS of the tryptic digest contained an m/z 4280 peak as predicted from the neurosecretory protein VGF precursor [Homo sapiens] sequence, and no m/z 4337 peak as predicted from the neuro-endocrine specific protein VGF [Homo sapiens] sequence.

**Figure 10:**
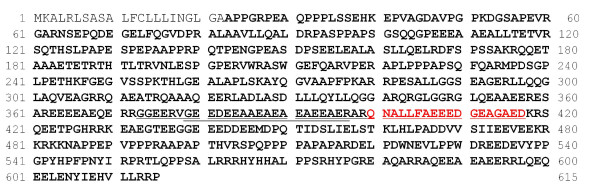
**Sequence of amino acids in neurosecretory protein VGF precursor [Homo sapiens]**. Bold: neurosecretory protein VGF(23-615). Red characters: MS/MS analysis (400-417, 1907.9 m/z)). Underline: analysis (373-417, 4808.8 m/z). Double underline: Carrette et al. (ref 13) confirmed 378-397 sequence from 4.8 kD peptide.

The 4.8-kDa CSF protein species was purified using anion ion exchange chromatography and reverse-phase high-performance liquid chromatography. The 4.8-kDa peak in the eluted fractions was detected using the Q10 ProteinChip array using the pH 8.0 binding/washing buffer. The 4.8-kDa protein eluted in the 100 and 200 mM NaCl fractions. The 100 and 200 mM NaCl fractions were purified by reverse phase high performance liquid chromatography (HPLC, TSK-GEL Super ODS, Toso, Tokyo, Japan). Fraction numbers 26 and 27 showed the 4.8-kDa peak protein and contained a predominant peak of 4.8 kDa between the 2000- to 10,000-Da range by SELDI analysis (Figures 5 and 6, data not shown). Protein species in reverse phase fractions 26 and 27 were concentrated and identified the protein by tandem mass spectrometry (MS/MS) of tryptic peptides. By single MS analysis, the m/z 1907 tryptic peptide was selected for MS/MS fragmentation and identification using the Mascot Search engine (Figure 7, 8). A Mascot search was performed on the fragmentation pattern from the 1907.8315 peak, and two protein candidates containing the QNALLFAEEEDGEAGAED peptide were identified, both with Mascot protein scores of 61. Because the 4.8-kDa protein was digested with trypsin to generate the m/z 1907 tryptic peptide, and the C-terminal amino acid of the identified peptide was not arginine or lysine as expected from trypsin cleavage, the QNALLFAEEEDGEAGAED sequence is best explained as the C-terminal region of the 4.8-kDa protein. This sequence was matched with equal Mascot scores to neurosecretory protein VGF precursor [*Homo sapiens*] and neuro-endocrine specific protein VGF [*Homo sapiens*]. With internal calibrants DynorphinA (2147.50) and human insulin (5807.65), an exact mass of the candidate peak was determined to have an m/z of 4808.35. Peptide mass fingerprint (PMF) analysis was used to distinguish between the two protein candidates identified by MS/MS by comparing predicted tryptic peptides less than 4.8 kDa with QNALLFAEEEDGEAGAED as the C-terminal sequence. MS of the tryptic digest revealed an m/z 4280 peak as predicted from the neurosecretory protein VGF precursor sequence, and no m/z 4337 peak as predicted from neuro-endocrine specific protein VGF sequence (Figure 9). It was therefore concluded that the 4.8-kDa protein is a proteolytic fragment of the neurosecretory protein VGF precursor (Figure 10).

## Discussion

Acute encephalopathy leads to rapid deterioration and a poor prognosis with neurologic complications. For detection of an early diagnostic marker for acute encephalopathy, SELDI-TOF MS was employed to identify possible biomarker candidates for acute encephalopathy in cerebrospinal fluid. A 4.8-kDa proteolytic peptide fragment from the protein of a neurosecretory protein VGF was identified as a novel biomarker for encephalopathy (VGF4.8). From previous literature, other peaks differentially expressed in the experiments in the present study were suggested to be: the double charge of Transthyretin (m/z 6880), which has neuroprotective functions [10]; Ubiquitin (m/z 8564), which is known to be involved in targeting proteins for degradation [11]; and Cystatin C 1-8 a truncated form (m/z 12536), which is a cysteine protease inhibitor and plays an important role in regulating extracellular protein homeostasis in the CNS [10,12]. The identification for these peaks is underway.

VGF4.8 and other similar VGF peptides have been reported in Alzheimer's disease [11,13-15], amyotrophic lateral sclerosis (ALS) [16], and frontotemporal dementia [17]. Carrette et al reported that VGEEDEEAAEAEAEAEEAER peptide, which was between the aminoacid 378 to 397 of neurosecretory protein VGF precursor, from the CSF in Alzheimer's disease [13]. This sequence was identical to our identified peptide sequence of VGF protein (Figure 10: double underline). The VGF4.8 peptides in CSF are decreased compared with normal controls in diseases such as Alzheimer's, ALS, and frontotemporal dementia. These diseases showed pathologically degenerative changes as well as encephalopathy, which were specifically investigated in this study. Thus, the VGF4.8 peptide might be a novel marker for degenerative brain conditions with encephalopathy in children.

VGF was first identified as a nerve growth factor (NGF)-regulated transcript in rat PC12 pheochromocytoma cells [16]. *In vivo*, the VGF mRNA and its encoded protein are selectively synthesized in neuroendocrine and neuronal cells. VGF is regulated by neuronal activity, including long-term potentiation, seizure, injury [13] and aging [18]. Brain-derived neurotrophic factor (BDNF) and NT-3 can also regulate the expression of VGF in cortical neurons [13]. In Alzheimer's disease, the BDNF mRNA is decreased. This decrease could result in a decrease of synthesis of the VGF precursor protein [13]. In similar situations, such as ALS and frontolateral dementia, progression of neuronal damage probably causes a decrease of neuronal products of VGF and loss of their protective function [14].

The primary VGF gene product is processed by endoproteolytic cleavages, resulting in a range of VGF peptides [16]. To date, 10 rat VGF peptides and three human VGF peptides have been identified [16]. The VGF4.8 sequence, which was obtained in this study, is rich in paired basic amino acid residues that are potential sites for proteolytic processing or sequence motifs of adjacent basic amino acids (arginine and/or lysine) as potential recognition sites for processing by prohormone convertases [14]. The modified compounds described in the present study are very likely biologically active and not merely incidental fragments [14].

It can be speculated that either deficiencies of neurotrophic factors or increased neurotrophic consumption in encephalopathy might lead to reduced levels of VGF [16]. Because VGF is essential for feeding behavior and energy expenditure, an enormous number of possible mechanisms whereby a deficiency of VGF might contribute to encephalopathy susceptibility or modify the phenotype of the clinical syndrome of encephalopathy are possible.

## Conclusions

We found a 4.8-kDa peptide fragment from a neurosecretory protein VGF precursor (VGF4.8) in CSF was identified as a novel biomarker for encephalopathy using Surface-enhanced laser desorption/ionization time-of-flight mass spectrometry (SELDI-TOF MS). Expression of VGF4.8 has been reported to be decreased in pathologically degenerative changes such as Alzheimer's disease, amyotrophic lateral sclerosis (ALS), and frontotemporal dementia. Thus the VGF4.8 peptide might be a novel marker for degenerative brain conditions. Further investigation into VGF4.8 could provide insight into the mechanism of encephalopathy as well as possible treatment strategies.

## List of abbreviation

ALS: amyotrophic lateral sclerosis; BDNF: brain derived neurotrophic factor; CSF: cerebrospinal fluid; EAM: energy-absorbing molecule; HPLC: reverse-phase high-performance liquid chromatography; MS: mass spectrometry; m/z: mass/charge ratio: NGF: nerve growth factor; PMF: peptide mass fingerprint; SELDI-TOF MS: surface-enhanced laser desorption/ionization time-of-flight mass spectrometry; SPA: saturated sinapinic acid; and VGF4.8: a 4.8-kDa proteolytic peptide fragment of neurosecretory protein VGF precursor.

## Competing interests

The authors declare that they have no competing interests.

## Authors' contributions

All authors read and approved the final version of manuscript.

TA had full access to all the data and take responsibility for the integrity of the data and the accuracy of the data analysis. Analysis and interpretation of data, drafting the manuscript, and the decision to the manuscript for publication were made by TA. Study concept and designwere contributed by TA and OF. Study supervision were done by TA, OF and YF. Collection of samples and data were done by SK, AT, TH, KK. Critical revision of the manuscript for important intellectual concept, obtained funding, administrative, technical and material support were done by OF and YF.

## Pre-publication history

The pre-publication history for this paper can be accessed here:

http://www.biomedcentral.com/1471-2377/11/101/prepub
